# Identification of genetic indicators linked to immunological infiltration in idiopathic pulmonary fibrosis

**DOI:** 10.1097/MD.0000000000042376

**Published:** 2025-05-09

**Authors:** Yan Huang, Yipei Ouyang, Wei Luo, Shiwen Huang

**Affiliations:** a School of Basic Medical Sciences, Youjiang Medical University for Nationalities, Baise, China; b Department of Neonatology, Affiliated Hospital of Youjiang Medical University for Nationalities, Baise, China; c Key Laboratory of Research on Clinical Molecular Diagnosis for High Incidence Diseases in Western Guangxi of Guangxi Higher Education Institutions, Baise, China; d Department of Cardiovascular Medicine, Affiliated Hospital of Youjiang Medical University for Nationalities, Baise, China.

**Keywords:** biomarker, CIBERSORT, idiopathic pulmonary fibrosis, immune infiltration, machine learning algorithm

## Abstract

This study employed bioinformatics to investigate potential molecular markers associated with idiopathic pulmonary fibrosis (IPF) and examined their correlation with immune-infiltrating cells. Microarray data for IPF were retrieved from the Gene Expression Omnibus database. Differentially expressed genes (DEGs) and module genes were identified through Limma analysis and weighted gene co-expression network analysis. Enrichment analysis and protein-protein interaction network development were performed on the DEGs. Machine learning algorithms, including least absolute shrinkage and selection operator regression, random forest, and extreme gradient boosting, were applied to identify potential key genes. The predictive accuracy was assessed through a nomogram and a receiver operating characteristic (ROC) curve. Additionally, the correlation between core genes and immune-infiltrating cells was assessed utilizing the CIBERSORT algorithm. An IPF model was established in a human fetal lung fibroblast 1 (HFL-1) through induction with transforming growth factor β1 (TGF-β1), and validation was conducted via reverse transcription-quantitative polymerase chain reaction. A sum of 1246 genes exhibited upregulation, whereas 879 genes were downregulated. Pathway enrichment analysis and functional annotation revealed that DEGs were predominantly involved in extracellular processes. Four key genes – *cd19*, *cxcl13*, *fcrl5*, and *slamf7* – were identified. Furthermore, ROC analysis demonstrated high predictive accuracy for these 4 genes. Compared to healthy individuals, lung tissues from IPF patients exhibited an increased presence of plasma cells, CD4 memory-activated T cells, M0 macrophages, activated dendritic cells, resting NK cells, and M2 macrophage infiltration. The upregulation of *cd19*, *cxcl13*, *fcrl5*, and *slamf7* in TGF-β1-treated HFL-1 cells was confirmed, aligning with the findings from the microarray data analysis. *cd19*, *cxcl13*, *fcrl5*, and *slamf7* serve as diagnostic markers for IPF, providing fresh perspectives regarding the fundamental pathogenesis and molecular mechanisms associated with this condition.

## 1. Introduction

Idiopathic pulmonary fibrosis (IPF) constitutes a rapidly progressive interstitial lung disease with a fatal prognosis. It is distinguished by persistent nonspecific interstitial inflammation and the excessive accumulation of collagen.^[1]^ The disease follows a gradual progression in its early stages, ultimately culminating in diffuse pulmonary fibrosis and mortality.^[2]^ The incidence of IPF ranges from 2 to 30 cases per 100,000 person-years, while its prevalence varies between 10 and 60 cases per 100,000 individuals.^[3]^ Following an IPF diagnosis, the median survival period is 3 to 5 years, with only 20% of patients remaining alive within this timeframe.^[4,5]^ Multiple risk factors contribute to the development of IPF, encompassing smoking, gastroesophageal reflux disease, viral infections, occupational or environmental exposure to inorganic dust (e.g., wood, metal, and silica dust), and air pollution.^[6]^ Moreover, the clinical prognosis for individuals with IPF remains poor. Currently, IPF has a restricted range of pharmaceutical interventions accessible for treatment. Among them, pirfenidone and nidanib have displayed efficacy but are associated with significant adverse effects. Therefore, identifying novel diagnostic biomarkers for IPF is imperative to advancing therapeutic strategies and improving patient outcomes.

Recent cancer research has increasingly focused on the infiltration of immune cells into tumors, attracting considerable attention. Pneumofibrosis has also been linked to immune cell activity.^[7]^ The stimulation of fibroblasts, angiogenesis, and connective tissue proliferation is mediated by inflammatory cytokines secreted by immune cells.^[8]^ Macrophages are recognized as key contributors to airway remodeling and pulmonary fibrosis.^[9]^ A more comprehensive understanding of immune cell infiltration in IPF is required.

Two datasets related to IPF were integrated from the Gene Expression Omnibus (GEO) database to establish a unified metadata collection. By analyzing this metadata cohort, distinct gene expression patterns were identified in tissues derived from individuals diagnosed with IPF in comparison to those obtained from healthy subjects. Functional annotation and enrichment analysis of the differentially expressed genes (DEGs) were conducted utilizing the Kyoto Encyclopedia of Genes and Genomes (KEGG) and Gene Ontology (GO) pathways. The distribution of immune cells within lung tissue from both IPF and non-IPF patients was assessed through the CIBERSORT algorithm. Genes associated with immune cells were examined using weighted gene co-expression network analysis (WGCNA). Afterward, potential gene biomarkers for IPF were identified through machine learning techniques. The diagnostic performance was evaluated using nomograms and receiver operating characteristic (ROC) curves. These findings may provide insights into enhancing IPF diagnosis and therapeutic strategies. Reverse transcription-quantitative polymerase chain reaction (RT-qPCR) analysis validated their upregulation in transforming growth factor β1 (TGF-β1)-treated human fetal lung fibroblast 1 (HFL-1).

## 2. Methods and materials

### 2.1. Collecting and processing data

Two gene expression datasets were procured from the GEO database (https://www.ncbi.nlm.nih.gov/geo/, last accessed on July 15, 2023): GSE110147 (GPL6244 Platform) and GSE53845 (GPL6480 Platform). Both datasets pertain to Homo sapiens.The GSE110147 dataset consisted of 22 lung tissue specimens from individuals diagnosed with IPF and 11 tissue samples adjacent to lung cancer resections, whereas the GSE53845 dataset encompassed 40 lung tissue specimens from individuals with IPF and 8 lung tissue specimens from healthy individuals. The SangerBox platform (https://sangerbox.com/, last accessed on July 15, 2023) was utilized to normalize the median gene expression data. COMBAT, an empirical Bayesian method, was subsequently employed to eliminate batch effects.^[10]^ The GEO dataset, as a publicly available dataset, does not necessitate a theoretical review.

### 2.2. DEG screening

First, the GSE110147 and GSE53845 datasets were combined and categorized into disease and normal groups. Principal component analysis (PCA) was implemented to reduce the dimensionality of the dataset using the SangerBox platform (https://sangerbox.com/; last accessed on July 15, 2023). The samples were subsequently clustered, and aberrant samples (GSM1302034 and GSM1302071) were excluded utilizing the dendrogram package in R (version 4.3.1). Subsequently, DEGs were identified per the criteria of |log_2_Fold change| >1.5 and adj. *P* < .05. The Limma analysis tool within the SangerBox platform was employed for statistical analysis and visualization.

### 2.3. An analysis of the enrichment of GO and KEGG

The Xiantao platform (https://www.xiantaozi.com/, last accessed on July 15, 2023) was utilized to perform GO and KEGG enrichment analyses of DEGs. GO analysis comprises 3 principal categories: Molecular function (MF), cellular components (CC), and biological processes (BP). KEGG was employed to examine the signaling pathways associated with the enriched DEGs. Statistically significant DEGs were identified per the threshold of *P *< .05 and a count ≥ 10. A bubble diagram was generated using an online application (https://www.xiantaozi.com/, last accessed on July 15, 2023) specifically designed for data processing and visualization.

### 2.4. Analyses of immune infiltration

The CIBERSORT web-based analysis tool was utilized to evaluate the gene expression profile of immune cell infiltration, thereby quantifying the distribution of immune cells in both the IPF and control groups.^[11]^ The Wilcoxon rank-sum test and statistical analysis methods were applied to assess disparities in immune cell composition between the 2 groups, and the results were compared using an online platform (https://www.xiantaozi.com/, last accessed on July 15, 2023).

### 2.5. Co-expression network construction

To further identify co-expression modules of core genes associated with immune cells, WGCNA was conducted. To identify a common expression module, the Oebiotech platform (https://cloud.oebiotech.com; last accessed July 15, 2023) was utilized. The following criteria were established: a standard deviation threshold of < 0.05 and a power value of 12 for network construction. The analysis yielded the following results: module identification and analysis, module trait-association analysis, and core gene analysis. The OECloud tool was employed for result visualization. For further examination, genes within the pink module exhibiting the highest association coefficient were selected.

### 2.6. Consistency cluster analysis

DEGs interact with immune infiltration-related genes within the pink module. A protein-protein interaction (PPI) network was developed utilizing the STRING database and subsequently visualized through Cytoscape. Consistency cluster analysis was performed utilizing the consistency cluster analysis tool available in Hiplot Pro (https://hiplot.com.cn/, last accessed on July 15, 2023), a comprehensive web-based platform designed for biomedical data analysis and visualization. Differential genes among IPF subtypes were identified through a differential analysis conducted using consistency cluster analysis, and the intersection of these differential genes with hub genes in patients was determined.

### 2.7. Machine learning

Three machine-learning approaches were employed to further identify candidate genes linked to IPF. The least absolute shrinkage and selection operator (LASSO) technique is a regression method that enhances the interpretability and predictive accuracy of statistical models.^[12]^ The random forest (RF) algorithm demonstrates high accuracy, specificity, and sensitivity, making it particularly suitable for predicting continuous variables without constraint.^[13]^ Regarding parallel computing efficiency, handling of missing values, and predictive performance, extreme gradient boosting (XGBoost) serves as a robust algorithmic toolkit based on the boosting framework.^[14]^ Online analysis tools (https://www.bioinformatics.com.cn, last accessed on July 15, 2023) and (https://hiplot.com.cn/, last accessed on July 15, 2023) were utilized as web-based platforms for data analysis and visualization. A potential hub gene for IPF was identified by integrating the results obtained from the 3 machine-learning methods.

### 2.8. Evaluation of the diagnostic value of candidate genes

The transcriptional patterns of the hub genes were assessed utilizing the GSE110147 and GSE53845 datasets. For the analysis and visualization of differential hub gene expression between the IPF and control groups, as well as between the 2 IPF subtypes and the control group, the HiPlot Pro platform (https://hiplot.com.cn/; last accessed on July 15, 2023) was employed. The area under the analytical curve (AUC), along with the 95% confidence interval, was determined. An AUC exceeding 0.7 signified excellent diagnostic performance.

### 2.9. Culture of human fetal lung fibroblasts 1

The HFL-1 was procured from Procell (CL-0106, WuHan, China). HFL-1 cells were maintained in Ham’s F-12K (L450 KJ; Basalmedia, ShangHai, China) medium comprising 10% fetal bovine serum (A5256701; Gibco, São Paulo, Brazil) and 1% Penicillin-Streptomycin (S110JV; Basalmedia) and were incubated at 37 °C in a humidified incubator with 5% CO₂. The experiment was categorized into a control group and a TGF-β1 group. In the TGF-β1 group, cells underwent exposure to TGF-β1 (10 ng/mL) (HY-P7118; MCE) for 24 hours to establish a pulmonary fibrosis cell model.

### 2.10. RT-qPCR

RNA was procured from cultured cells using Trizol (H10318; TransGen Biotech, BeiJing, China). Total RNA underwent reverse transcription to complementary DNA utilizing the BeyoRT™ II First Strand cDNA Synthesis Kit (D7168M; Beyotime, ShangHai, China). qPCR was executed with the SuperStar Universal SYBR Master Mix (CW3360M; cowin, JiangSu, China). Primers were synthesized by GENCEFE Biotech (JiangSu, China), with GAPDH serving as the internal reference, and relative gene expression levels were ascertained utilizing the 2^−ΔΔCT^ method. The following primers were used for qPCR:human GAPDH-F:5′-GAT TTG GTC GTA TTG GGC GC-3′,human GAPDH-R:5′-AGT GAT GG CAT GGA CTG TGG-3′;human CD19-F:5′-GAC AGT CAA TGT GGA GGG CA-3′,human CD19-R:5′-CCA TAG TAC TGG CCG AGC AG-3′;human CXCL13-F:5′-GTG TGG ACC CTC AAG CTG AA -3′,human CXCL13-R:5′-GGC TCA AGT TCC ATC TGC CT-3′;human FCRL5-F:5′-CAC GTC TCT CCA ACT GCT GT-3′,human FCRL5-R:5′-GGT GTC AAG TGC CGA CCT TA-3′;Human SLAMF7-F:5′-CCA ACA TGC CTC ACC CTC AT -3′,human SLAMF7-R:5′-GGG AAT GCA CTG CTG TCT AG-3.

### 2.11. Statistical analysis

All statistical analyses were conducted utilizing R software (version 4.2.1) and GraphPad Prism 9. Data were denoted as the mean ± standard deviation. Comparisons between 2 groups were executed using either the *t* test or the Wilcoxon rank sum test, whereas a one-way analysis of variance was applied for comparisons among multiple groups. Statistical significance was determined at *P* < .05.

## 3. Results

### 3.1. Data preprocessing and differential gene screening

**Figure 1.**
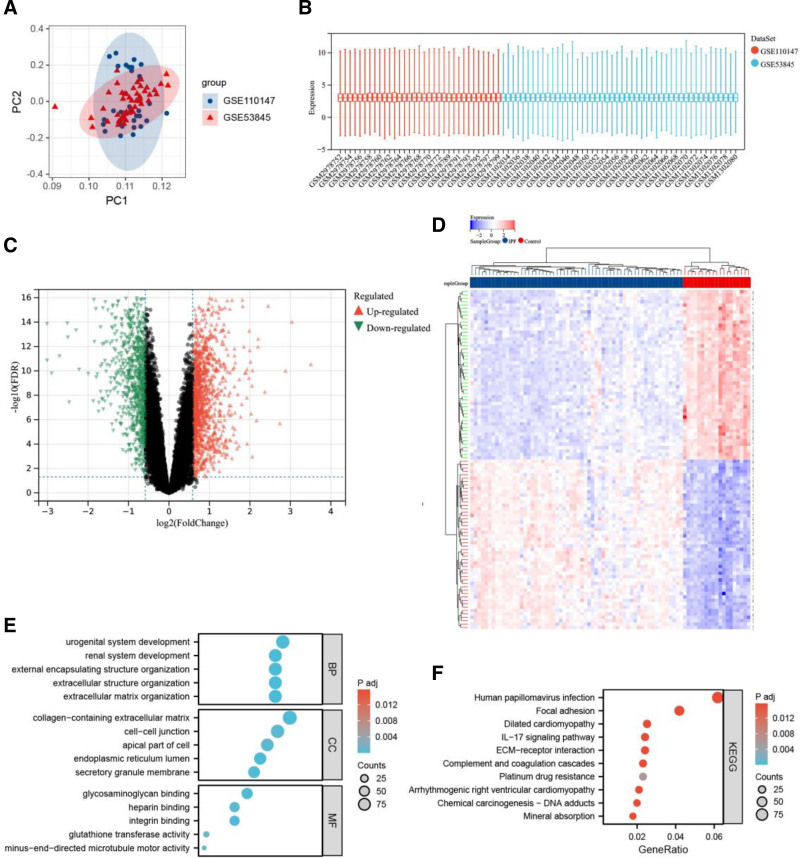
Differential gene expression analysis between IPF patients and healthy individuals following batch effect elimination. A. PCA visualization of both datasets subsequent to batch effect removal. B. Distribution analysis represented through box plots post-batch effect elimination. C. Differential gene expression is illustrated through a volcano plot, wherein upregulated genes are depicted in red, downregulated genes in green, and non-DEGs between IPF patients and healthy controls are shown in black. D. Hierarchical clustering heatmap demonstrates the expression patterns of identified DEGs across sixty individuals diagnosed with IPF and nineteen healthy controls. Expression intensity is represented by a color gradient, with red indicating elevated expression and blue denoting reduced expression. E. GO enrichment analysis of identified DEGs. F. Top 10 markedly enriched KEGG pathways. DEGs = differentially expressed genes, GO = gene ontology, IPF = idiopathic pulmonary fibrosis, KEGG = Kyoto Encyclopedia of Genes and Genomes, PCA = principal component analysis.

First, the GSE110147 and GSE53845 datasets were standardized and integrated to mitigate batch effects, with the results visualized using PCA plots (Fig. 1A) and box plots (Fig. 1B). The PCA results (Figure S1, Supplemental Digital Content, https://links.lww.com/MD/O856) demonstrated improved clustering of the 2 sample groups following normalization, confirming the reliability of the sample source. Additionally, the box plot results indicated the successful elimination of inter-batch differences. Outlier samples GSM1302034 and GSM1302071 were removed, as illustrated in the hierarchical clustering diagram (Figure S2, Supplemental Digital Content, https://links.lww.com/MD/O856). A sum of 2125 DEGs was identified, among which 1246 were elevated (log₂FC ≥ 1), and 879 were diminished (log₂FC ≤ -1). The expression patterns of these DEGs are depicted in volcano and heat maps (Fig. 1C and D). GO analysis indicated that DEG-associated BP was primarily enriched in urogenital system development, renal system development, outer envelope structure organization, extracellular matrix (ECM) organization, and extracellular structural components. Furthermore, the CC of DEGs was markedly enriched in ECM-containing collagen, the endoplasmic reticulum lumen, the apical portion of cells, and secretory granules. MF enrichment analysis highlighted ECM structural components, integrin binding, heparin binding, glutathione transferase activity, and negative end-directed microtubule motility activity. The top 10 GO terms ranked according to the adjusted *P* value are displayed in Figure 1E. According to KEGG pathway analysis based on the adjusted *P* value, the top 10 DEG-enriched pathways were predominantly associated with human papillomavirus infection, focal adhesion, dilated cardiomyopathy, interleukin-17 signaling, the complement and coagulation cascade, platinum resistance, arrhythmogenic right ventricular cardiomyopathy, and the mineral absorption pathway (Fig. 1F).

### 3.2. Results of immune cell infiltration

**Figure 2.**
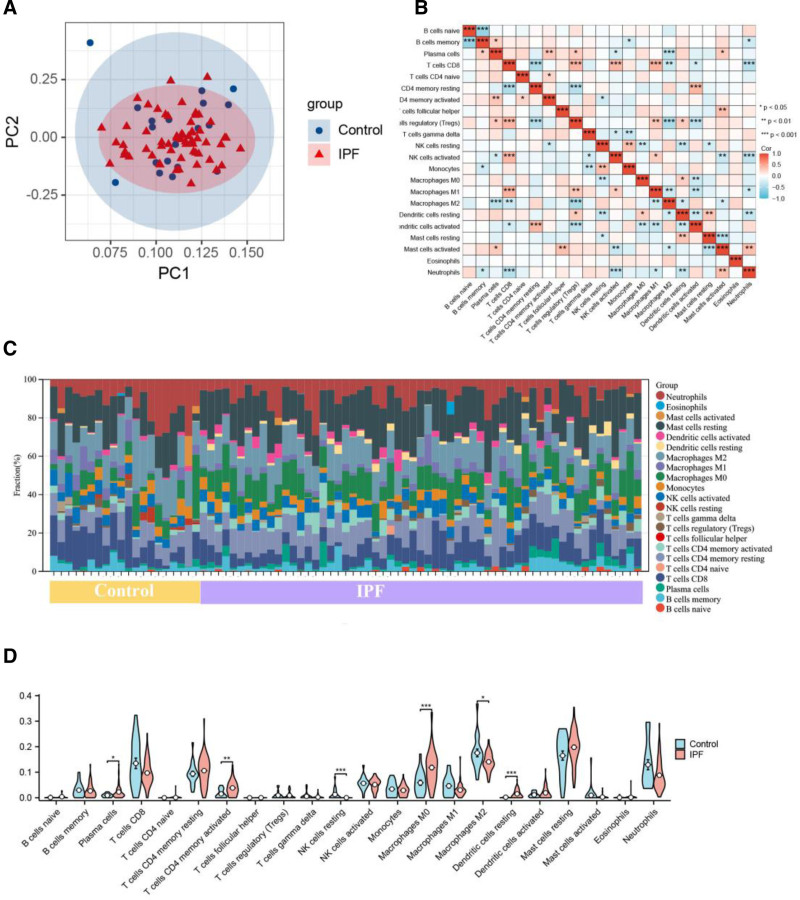
Immune cell infiltration in IPF and controls. A. PCA clustering visualization demonstrating immune cell infiltration distribution between IPF and control specimens. B. Correlation matrix heat map depicting the interrelationships among 22 distinct immune cell populations. C. Quantitative distribution of 22 immune cell infiltration subtypes, with the X-axis representing relative percentages across 19 control and 60 IPF samples, while the Y-axis indicates the proportional composition of immune cell subtypes per specimen (color-coded classification of immune cell categories displayed on the right). D. Statistical visualization via violin plots illustrating differential immune cell infiltration patterns between IPF and healthy control groups across 22 immune cell populations. *P < .05; **P < .01; ***P < .001. IPF = idiopathic pulmonary fibrosis, PCA = principal component analysis.

PCA cluster analysis was employed to examine the consistency and discrepancies between groups. The results of PCA cluster analysis suggested statistically significant differences in immune cell infiltration between IPF patients and controls (Fig. 2A). The correlation heat map of the 22 immune cell types illustrated the relationships among these immune cells (Fig. 2B). The proportions of the 22 immune cell subsets demonstrated that Macrophages M0, resting Mast cells, Neutrophils, Macrophages M2, and CD4 memory resting T cells exhibited the most pronounced differences in infiltration between the IPF and control groups (Fig. 2C). The violin plot depicting immune cell infiltration differences indicated an increased presence of plasma cells, CD4 memory activated T cells, Macrophages M0, and activated Dendritic cells, whereas a diminished infiltration of resting NK cells and Macrophages M2 was observed in comparison to normal control samples (Fig. 2D).

### 3.3. WGCNA was conducted to identify key modules and functional enrichment analysis

**Figure 3.**
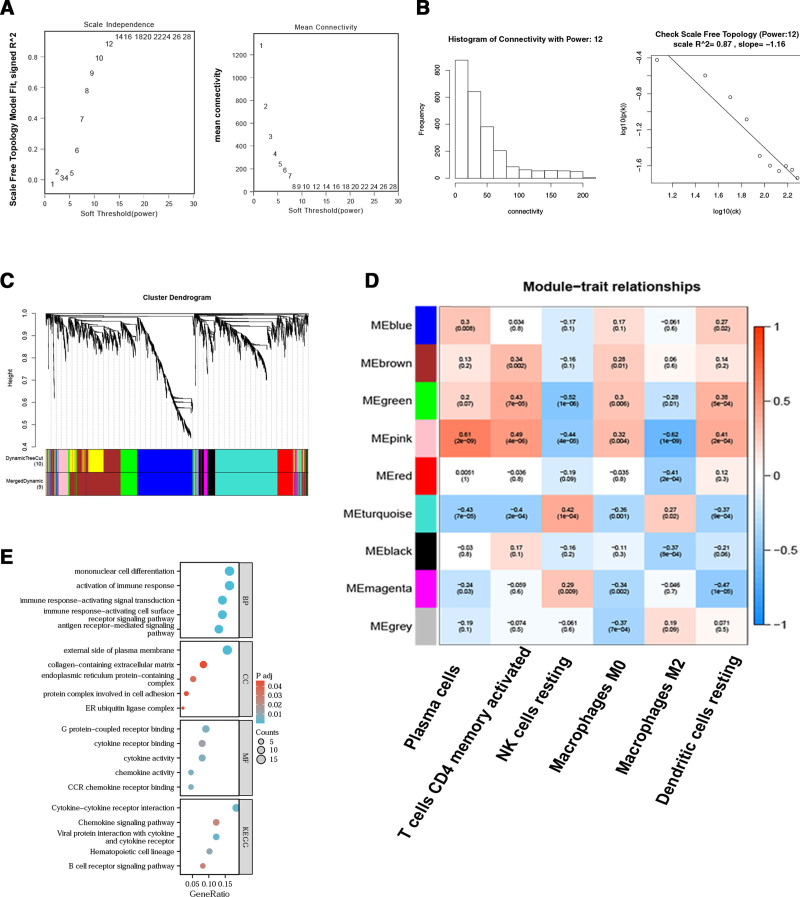
WGCNA for identification of module genes exhibiting correlation with immune infiltration. (A) and (B) Evaluation of diverse soft thresholds for network topology optimization (A). The x-axis represents the power value, while the y-axis of the left and right graphs depict network correlation coefficients and average network connectivity, respectively; in the right graph, a progressive reduction in average gene connectivity is observed with increasing power values. B. Distribution histogram illustrating connectivity metrics relative to power values with scale-free topological assessment. C. Hierarchical dendrogram visualizing color-coded co-expression gene modules. D. Correlation matrix depicting associations between distinct gene modules and immune cell populations. E. Functional enrichment analysis performed on genes within the pink module. WGCNA = weighted gene co-expression networks.

WGCNA was employed to identify module genes exhibiting the strongest correlation with immune cells in IPF. Based on a scale independence value exceeding 0.8, a soft threshold power (β) of 12 was selected to ensure the biological relevance of the scale-free network (Fig. 3A and B). A clustering dendrogram of IPF and control samples was constructed according to scale independence and average connectivity, resulting in 9 distinct gene co-expression modules, each designated by a unique color (Fig. 3C). Among these, the pink module exhibited the highest correlation with immune cells and was selected for further analysis (Fig. 3D, *P* < .05). A sum of 103 genes within the pink module were analyzed for functional enrichment. GO analysis demonstrated significant enrichment of BP, including single-cell differentiation, immune response activation, receptor signaling pathways, and antigen receptor-mediated signaling pathways. In the CC category, enriched genes were associated with the outer plasma membrane, ECM, endoplasmic reticulum, protein complexes involved in cellular adhesion, and ER ubiquitin ligase complexes. MF enrichment analysis indicated significant involvement in G protein-coupled receptor binding, cytokine receptor binding, cytokine activity, chemokine activity, and CCR chemokine receptor binding. KEGG pathway analysis revealed that the most highly enriched genes within the pink module were implicated in cytokine–cytokine receptor interactions, chemokine signaling pathways, cytokine-receptor interactions, viral protein-associated pathways, and hematopoietic cell lineage differentiation. Additionally, B cell receptor signaling pathways were identified as markedly enriched (Fig. 3E). Overall, the biological functions of the pink module were closely associated with immune cell activity.

### 3.4. Consistency cluster analysis

**Figure 4.**
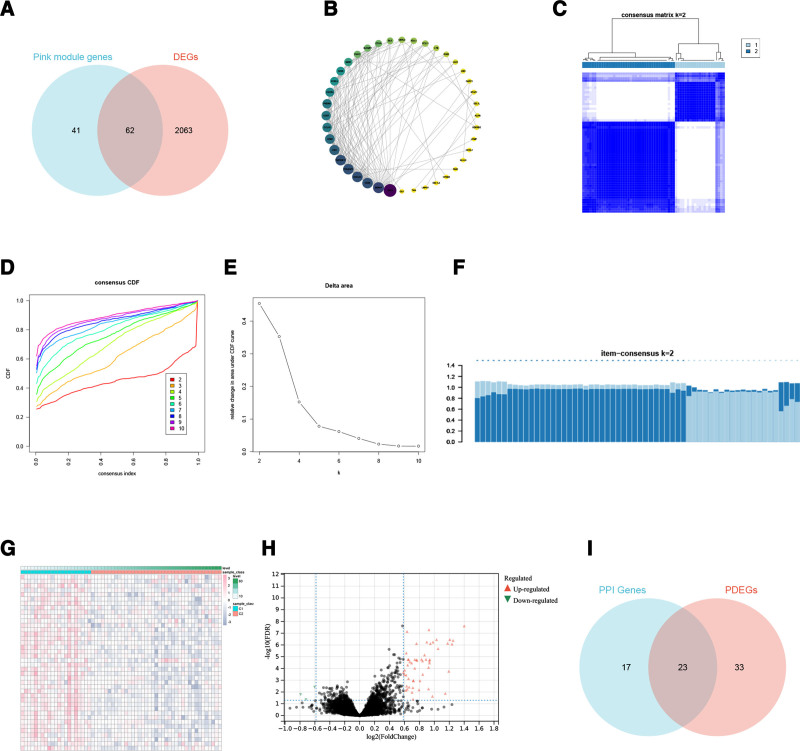
Disease typing of IPF identified by consistent cluster analysis. A. Genes positioned at the intersection of immune cell-associated genes and DEGs. B. PPI network rendered via Cytoscape software. C. Consensus matrix visualization for IPF molecular subtypes. D. cumulative distribution function curve of IPF consensus clustering. E. Relative alterations in the underlying cumulative distribution function curve for IPF subtyping. F. Item-consensus plot with k = 2, demonstrating optimal stratification parameters. G. Heatmap visualization of gene expression patterns across identified IPF subtypes following consensus clustering. H. Volcano plot illustrating differential gene expression profiles between distinct IPF patient subtypes. Genes exhibiting upregulation are depicted in red, those demonstrating downregulation are represented in green, and genes lacking significant differential expression between IPF phenotypes are shown in black. I. Intersection of patient-derived hub genes and DEGs across IPF subtypes. DEGs = differentially expressed genes, IPF = idiopathic pulmonary fibrosis, PPI = protein-protein interaction.

Genes associated with immune cells were intersected with DEGs identified through WGCNA, resulting in 62 intersecting genes (Fig. 4A). The STRING database was utilized to construct a PPI network, which was subsequently visualized using Cytoscape. After the removal of genes lacking node connections, 40 genes remained for visualization (Fig. 4B). Consensus clustering was conducted on IPF samples based on these 40 intersecting genes, and the optimal classification parameter for patient stratification was determined to be K = 2, as indicated by the consensus matrix, consensus cumulative distribution function, relative change under the cumulative distribution function curve, and item consensus analysis (Fig. 4C–F). Following consensus clustering, a heatmap was generated to illustrate differences in gene expression among IPF patients (Fig. 4G). Based on differential gene expression profiling after clustering, 100 DEGs were identified according to the adjusted *P*-value threshold (<0.05) and |log₂FC| > 1.5. The expression patterns of these DEGs are depicted in a volcano plot (Fig. 4H). Additionally, 23 overlapping genes were identified by intersecting the genes obtained from PPI network visualization with those exhibiting differential expression among the identified patient subtypes (Fig. 4I).

### 3.5. Identifying candidate hub genes by machine learning

**Figure 5.**
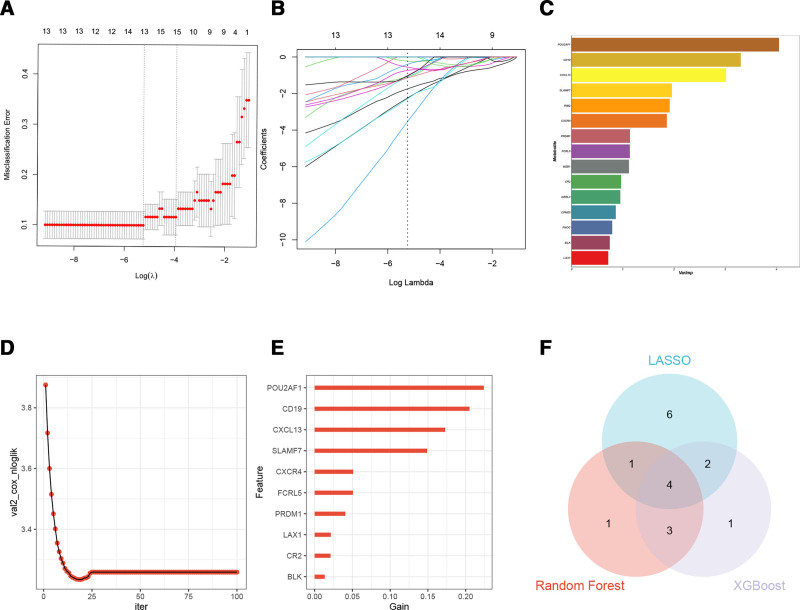
Screening candidate diagnostic biomarkers for IPF using machine learning. (A) and (B) Implementation of LASSO logistic regression algorithm for diagnostic marker screening and selection. C. Graphical representation displaying the 10 most significant genes as determined by the Mean Decrease Gini criterion within the RF algorithm framework. (D) and (E) Systematic biomarker identification process utilizing XGBoost regression modeling techniques. F. Intersectional Venn diagram illustrating diagnostic markers (*cd19*, *cxcl13*, *fcrl5*, *slamf7*) commonly identified across 3 distinct machine learning algorithmic approaches. LASSO = least absolute shrinkage and selection operator, RF = random forest.

Diagnostic indicators associated with disease diagnosis were identified using the RF, LASSO, and XGBoost regression models. As illustrated in Figure 5A and B, 13 diagnostic core genes of IPF were selected from DEGs through the LASSO regression algorithm. The RF model identified 9 diagnostic core genes for IPF (Fig. 5C). Similarly, the XGBoost regression model was employed to screen 10 diagnostic core genes for IPF (Fig. 5D and E). By integrating the outcomes from the 3 machine-learning algorithms, 4 common diagnostic markers of IPF were identified: *cd19*, *cxcl13*, *fcrl5*, and *slamf7* (Fig. 5F).

### 3.6. Diagnostic value assessment

**Figure 6.**
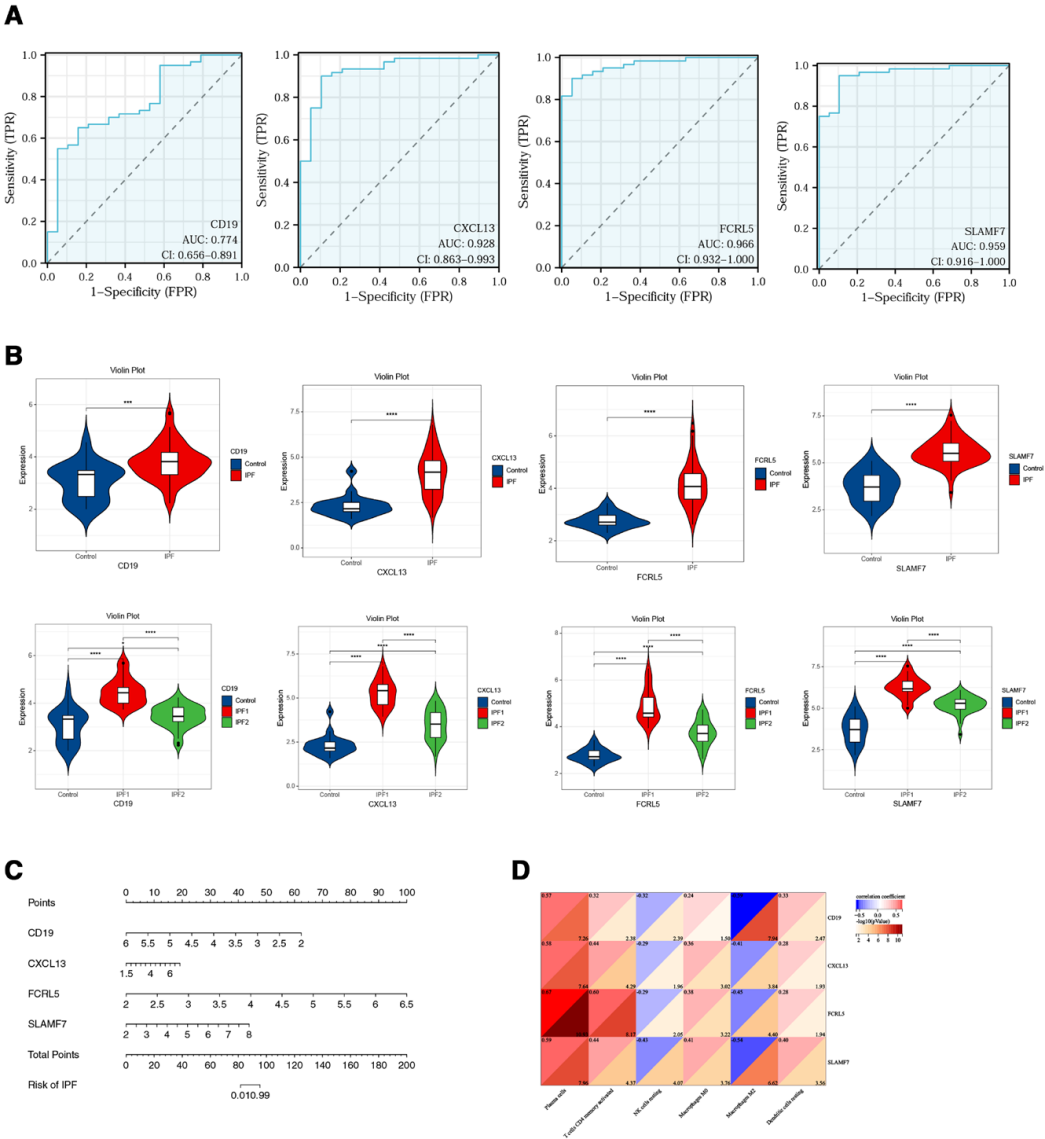
Validation of the diagnostic value of *cd19*, *cxcl13*, *fcrl5,* and *slamf7* as hub genes. A. ROC curve for diagnostic effectiveness validation. B. Violin plot showing the expression levels of *cd19*, *cxcl13*, *fcrl5*, and *slamf7* between IPF group and control group, IPF group phenotypes and controls. C. Nomogram predicting the probability of IPF. D. Relevance of 4 key genes to immune cells. IPF = idiopathic pulmonary fibrosis, ROC = receiver operating characteristic.

ROC logistic regression analysis was conducted to evaluate the diagnostic utility of the 4 hub genes in IPF. An AUC value exceeding 0.700 was considered indicative of strong diagnostic reliability. The results demonstrated AUC values of 0.774 for *cd19*, 0.928 for *cxcl13*, 0.966 for *fcrl5*, and 0.959 for *slamf7*, suggesting that these 4 hub genes exhibited high sensitivity and specificity in the diagnosis of IPF (Fig. 6A). Expression levels of *cd19*, *cxcl13*, *fcrl5*, and *slamf7* were markedly elevated in the IPF group versus the control group (*P* < .001). Furthermore, expression levels in the IPF1 subgroup were markedly higher than those in both the control group and the IPF2 subgroup (*P *< .0001) (Fig. 6B). To facilitate clinical decision-making, nomograms were constructed to provide a quantitative approach for predicting disease risk (Fig. 6C). Correlation analysis revealed that plasma cells exhibited the highest correlation with *cxcl13*, *fcrl5*, and *slamf7*, whereas Macrophages M2 demonstrated the strongest correlation with *cd19* (Fig. 6D).

### 3.7. Preliminary validation of hub gene expression in fibroblasts

**Figure 7.**
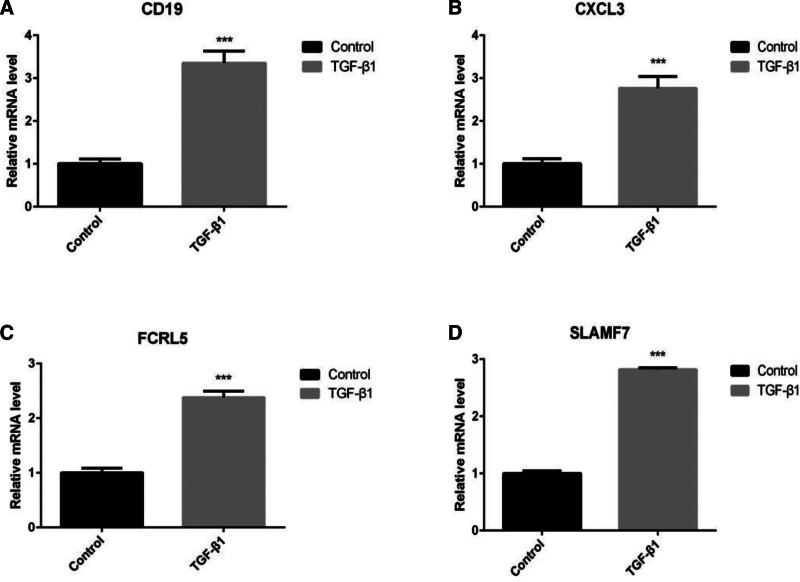
Verification of differential gene expression in the TGF-β1-induced pulmonary fibrosis HFL-1 cell model. (A–D) Relative mRNA expression levels of *cd19*, *cxcl13*, *fcrl5*, and *slamf7* were quantified via RT-qPCR analysis. (n = 3 per group). *P < .05; **P < .01; ***P < .001. HFL-1 = Human Fetal Lung Fibroblast 1, RT-qPCR = Reverse Transcription Quantitative Polymerase Chain Reaction, TGF-β1 = transforming growth factor β1.

To validate the bioinformatics findings, the relative mRNA expression levels of *cd19*, *cxcl13*, *fcrl5*, and *slamf7* in TGF-β1-treated HFL-1 cells were evaluated through RT-qPCR. TGF-β1, a well-established profibrotic factor, was employed to induce fibroblast activation. A significant upregulation in the mRNA expression of these 4 genes was observed in the TGF-β1-treated group versus the control group (*P* < .001) (Fig. 7A–D).

## 4. Discussion

The etiology of IPF, a chronic, progressive, and ultimately fatal lung disease, is multifactorial, encompassing a range of contributing factors. In this pathological state, abnormal scarring of the alveolar epithelium occurs, leading to the excessive accumulation of ECM. Fibroblasts and myofibroblasts, which constitute fibroblastic foci, exhibit an overproduction of ECM.^[15]^

Due to the lack of early detection markers for IPF, patients frequently fail to receive treatment within the optimal time frame, resulting in worsening conditions. Thus, the identification of therapeutic targets and the exploration of molecular mechanisms linked to biomarkers associated with IPF are essential. Inflammatory cells have also been recognized as contributors to fibrosis.^[16]^ Examining the relationship between immune-infiltrating cells and genes implicated in IPF may improve the condition’s prognosis.

Two datasets, GSE110147 and GSE53845, were retrieved from the GEO database and subsequently merged to facilitate a comprehensive analysis. A total of 2125 DEGs were identified, with 1246 showing elevated expression levels and 879 exhibiting reduced expression levels in IPF. The functional roles of these DEGs were examined through GO and KEGG enrichment analyses, which corroborated findings from previous mechanistic studies. Biological functions of the DEGs included processes such as the development of the urogenital and kidney systems, the organization of the outer encapsulation structure, ECM assembly, and extracellular structure organization. Components such as collagen-containing ECM, the endoplasmic reticulum lumen, the apical portion of cells, secretory granzyme, integrin binding, heparin-binding, and glutathione transferase activity/negative end-directed microtubule motility were identified. These differentially expressed ECM functions indicate their possible contribution to the pathogenesis and progression of IPF. KEGG pathway enrichment analysis demonstrated that DEGs were markedly associated with pathways related to human papillomavirus infection, focal adhesion, dilated cardiomyopathy, the interleukin-17 signaling pathway, complement and coagulation cascades, platinum resistance, arrhythmic right ventricular cardiomyopathy, and mineral absorption, among others. These findings align with earlier research that suggested a role for cytokine release and inflammatory responses in the onset and progression of IPF.

Using the LASSO regression model, RF, and XGBOOST analysis, *cd19*, *cxcl13*, *fcrl5,* and *slamf7* were identified as diagnostic markers for IPF. These results were consistent with those from microarray analysis, and RT-qPCR confirmed that these 4 genes were highly expressed in a TGF-β1-induced HFL-1 cell lung fibrosis model.*cd19*, a B cell-specific member of the immunoglobulin superfamily, is expressed by pre-B cells that have undergone heavy-chain rearrangement and continue to be expressed until early plasma cell differentiation.^[17,18]^ This 95-kDa type I membrane glycoprotein^[19]^ is broadly distributed on the surface of B cells. In vitro studies have demonstrated that *cd19* serves a pivotal function in regulating B cell growth and development,^[20,21]^ as well as in modulating B cell activation, proliferation, and signaling. B cells are critically involved in the immune response and fibrosis associated with IPF.^[22]^ As a key marker of B cells, *cd19* may participate in this immunomodulatory network, influencing both inflammatory progression and fibrotic processes. Evidence has indicated that the development of pulmonary fibrosis is linked to the *cd19* signaling pathway, which modulates B cell infiltration into lung tissue.^[23]^ Through signaling via Toll-like receptor 4, *cd19* has been shown to regulate the production of fibrotic cytokines by B cells and to control fibrotic responses in both skin and lung tissues.^[24]^ These findings underscore the significance of *cd19* in regulating B cell function and immune responses while mitigating inflammation and fibrosis in IPF. *cxcl13*, a member of the C-X-C chemokine family, is a small molecular protein capable of binding to specific receptors to exert its biological functions.^[25,26]^ As a B-cell chemokine, *cxcl13* facilitates the aggregation of B cells toward inflammatory sites. It is synthesized in autoimmune diseases and has been implicated in inflammatory processes.^[27]^ Macrophages and dendritic cells derived from follicles in secondary lymphoid organs have been identified as major sources of *cxcl13*, which binds to CXC chemokine receptor 5. By activating this receptor, *cxcl1*3 serves a crucial function in modulating immune responses by guiding B and T cell homing.^[28]^ Studies have revealed that plasma *cxcl13* concentrations are closely correlated with the progression and severity of IPF, making it a potential biomarker for predicting disease trajectory,^[29]^ this is consistent with the results of our study. Elevated levels of *cxcl13* have been detected in the blood of IPF patients, with even higher serum concentrations observed in those experiencing non-arterial hypertension or acute exacerbations.^[30]^ Furthermore, *cxcl13* has been implicated in numerous inflammatory conditions,^[31,32]^ autoimmune disorders,^[33]^ and malignancies.^[34]^ Its involvement in diverse pathological states suggests that *cxcl13* may serve as a promising therapeutic target. *Fcrl5* is a transmembrane glycoprotein characterized by structural domains belonging to the immunoglobulin superfamily, which allow interactions with immunoglobulins such as IgG. This protein is predominantly expressed in B cells^[35]^ and has been reported to promote B cell proliferation and differentiation.^[36]^ *Fcrl5* exhibits strong evolutionary conservation and functions as a multifunctional factor involved in various BP, including transcription, DNA repair, cellular differentiation, developmental events, and extracellular signaling.^[37]^ Aberrant expression of *Fcrl5* has been associated with multiple autoimmune and infectious diseases.^[38]^ In multiple myeloma, its upregulation has been linked to disease progression.^[39]^ Although its role in pulmonary fibrosis has not been extensively reported, *Fcrl5*-positive B cells have been identified in the bronchoalveolar lavage fluid of IPF patients.^[40]^ The presence of these cells suggests their involvement in the immune responses associated with disease progression. Their abnormal accumulation and functional alterations within lung tissue may contribute to the persistent inflammatory response and tissue repair mechanisms characteristic of IPF. *slamf7* is a transmembrane receptor protein with a molecular weight of approximately 66 kDa, classified within the immunoglobulin superfamily. Two major isoforms of the *slamf7* protein, *slamf7*-long and *slamf7*-short, have been identified. Under physiological conditions, *slamf7* is expressed at low levels in various immune cell populations, including partially activated T and B cell subsets, dendritic cells, monocytes/macrophages, and plasma cells.^[41,42]^ Previous research has indicated that *slamf7* expression is markedly elevated in B cells from rheumatoid arthritis-associated interstitial lung disease patients compared to those with RA alone. Moreover, increased *slamf7* protein expression has been detected in the plasma of rheumatoid arthritis-associated interstitial lung disease patients, suggesting a potential role for *slamf7* in the pathogenesis and progression of pulmonary fibrosis.^[43]^ Additionally, studies have demonstrated that ALKBH5 can modulate the autophagic function of macrophages by regulating the m6A modification of *slamf7*, thereby influencing silica-induced lung inflammation.^[44]^ This evidence suggests that *slamf7* may contribute to pulmonary fibrosis through ALKBH5-associated signaling pathways and may represent a novel therapeutic target. In this study, bioinformatics methods were employed to identify DEGs. Some of these genes have been previously confirmed to be associated with the pathogenesis of IPF, whereas others have yet to be thoroughly investigated. The unexplored genes identified in this analysis may serve as novel research targets for further elucidation of the molecular mechanisms underlying IPF.

The CIBERSORT method was employed to evaluate immune cell infiltration in individuals diagnosed with IPF and in healthy controls. Multiple immune cell subtypes have been implicated in the pathogenesis and progression of IPF. Compared to healthy controls, increased levels of M0 macrophages, resting dendritic cells, plasma cells, and CD4 memory-activated T cells were observed in IPF patients, whereas lower levels of resting NK cells and M2 macrophages were detected. Consequently, the involvement of these immune cell populations in the pathogenesis and progression of IPF appears plausible. Furthermore, correlation analyses between *cd19*, *cxcl13*, *fcrl5*, *slamf7*, and immune cells revealed associations between *cd19*, *cxcl13*, *fcrl5*, and *slamf7* with M2 macrophages and plasma cells. The progression of IPF is largely influenced by chronic inflammation and immune dysregulation, as immune responses have been strongly associated with the disease. Macrophages serve a fundamental function in immune defense and tissue repair mechanisms.

In fibrotic lungs, M2 macrophages are widely present.^[45]^ These macrophages are known to secrete multiple growth factors, including fibroblast growth factor, insulin-like growth factor 1, transforming growth factor beta, and vascular endothelial growth factor.^[46]^ Additionally, they actively contribute to ECM formation and serve a pivotal function in the regulation of fibrosis. Neutrophils serve as the primary responders to tissue injury or pathogen invasion within the human lungs. Functioning in concert with lymphocytes and other granulocytes, neutrophils facilitate lung tissue inflammation and participate in both innate and adaptive immune responses. A major product of neutrophils, neutrophil elastase, has been implicated in the hydrolysis of bronchial tissue, the degradation of various ECM components, and the damage to alveolar epithelial and capillary endothelial cells. These pathological effects disrupt alveolar architecture and ultimately contribute to the progression of pulmonary fibrosis.^[47]^

According to a study, a markedly higher number of eosinophils has been detected in the bronchoalveolar lavage fluid of patients with IPF.^[48]^ Additionally, peptidyl-prolyl isomerase derived from eosinophils has been shown to enhance the stability of TGF-β mRNA, thereby contributing to the progression of pulmonary fibrosis.^[49]^ Mast cells are also recognized as significant contributors to the progression of pulmonary fibrosis. In individuals diagnosed with IPF, fibrotic lung tissue has been found to contain increased numbers of mast cells, accompanied by elevated secretion levels of TGF-β within these cells. Accumulating evidence suggests a correlation between Th1/Th2 imbalance and the inflammatory phase of pulmonary fibrosis. The secretion of IFN-γ and IL-12 by Th1 cells has been shown to exert anti-fibrotic effects, whereas the production of IL-4, IL-5, and IL-13 by Th2 cells has been implicated in the progression of pulmonary fibrosis.^[50]^ NKT cells are regarded as key regulators in the pathogenesis of fibrotic diseases.^[51]^ These cells serve a vital function in immune system regulation, responding rapidly to stimuli and releasing substantial quantities of cytokines that amplify and modulate immune responses.^[52]^ As a principal source of Th1 and Th2 cytokines, NKT cells are thought to influence macrophage polarization during pulmonary fibrosis. Cytokines secreted by NKT cells may contribute to M1/M2 macrophage polarization, thereby affecting the initiation and progression of pulmonary fibrosis.^[53]^

In this study, the GSE110147 and GSE53845 datasets were integrated to investigate the molecular pathways involved in the pathogenesis of IPF based on DEGs. However, the precise molecular mechanisms underlying the pathogenesis of IPF within the identified DEGs require further investigation. Several limitations should be acknowledged in this study. First, the analysis was restricted to internal datasets, and external validation was not performed. Second, only in vitro experiments were conducted to verify gene expression levels, while validation through animal experiments was not included. Additionally, only mRNA expression levels were assessed, necessitating further verification through techniques such as Western blot, immunofluorescence, and immunohistochemistry. Lastly, the incorporation of advanced technologies and additional bioinformatics approaches is required to identify DEGs with greater accuracy. The present study utilized bioinformatics to analyze potential molecular factors implicated in the pathogenesis of IPF and preliminary experimental validation was performed, which provided a reliable perspective on the underlying pathogenesis of IPF.

## 5. Conclusion

In this study, 4 potential pivotal genes (*cd19*, *cxcl13*, *fcrl5*, and *slamf7*) associated with immunity were identified as possible diagnostic markers for IPF. The presence of plasma cells, M0 and M2 macrophages, neutrophils, and CD4 memory resting T cells was found to be closely linked to the development and progression of IPF. These findings indicate that immunotherapy targeting immune-related factors may represent a promising approach for IPF treatment.

## Acknowledgments

We extend our sincere gratitude to GEO, Sanger Box, Hiplot Pro, Oebiotech, and Shanghai New Core Biotechnology for generously providing their platforms. We also appreciate the contributors for uploading valuable datasets that made this study possible.

## Author contributions

**Data curation:** Yan Huang.

**Investigation:** Wei Luo.

**Methodology:** Yan Huang, Wei Luo, Yipei Ouyang.

**Supervision:** Shiwen Huang.

**Validation:** Yan Huang.

**Visualization:** Yan Huang, Yipei Ouyang.

**Writing – original draft:** Yan Huang, Yipei Ouyang.

**Writing – review & editing:** Yan Huang, Shiwen Huang.

## Supplementary Material


