# Supplementary material for: Identification of genetic indicators linked to immunological infiltration in idiopathic pulmonary fibrosis
Source: Medicine (Baltimore). 2025 May 9;104(19):e42376. doi: 10.1097/MD.0000000000042376 (PMC12073941; doi:10.1097/MD.0000000000042376)
Supplement: Supplementary file 1 [file medi-104-e42376-s001.docx]

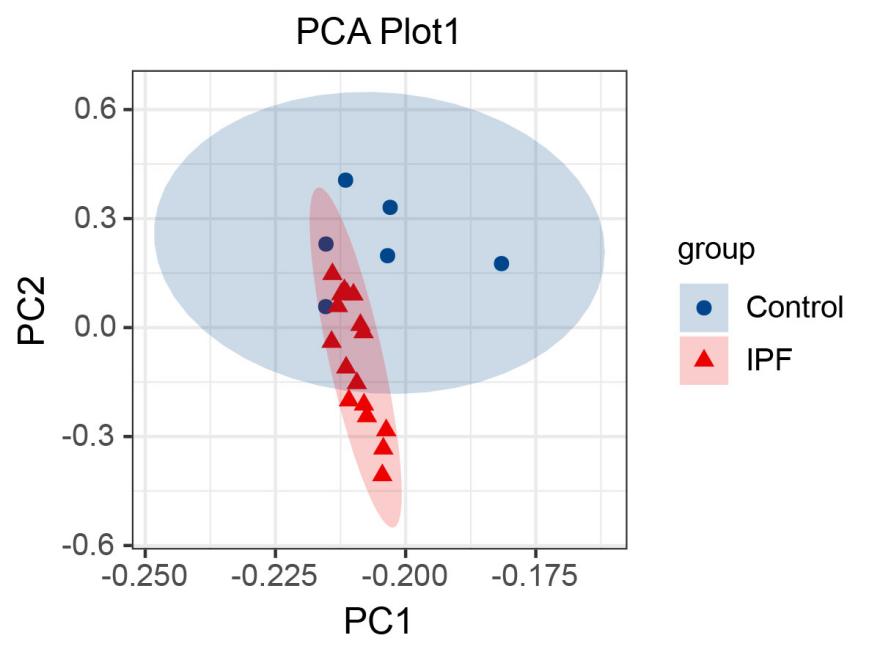


Figure Supplementary 1 PCA plots of the two sets of samples after removal of batch effects


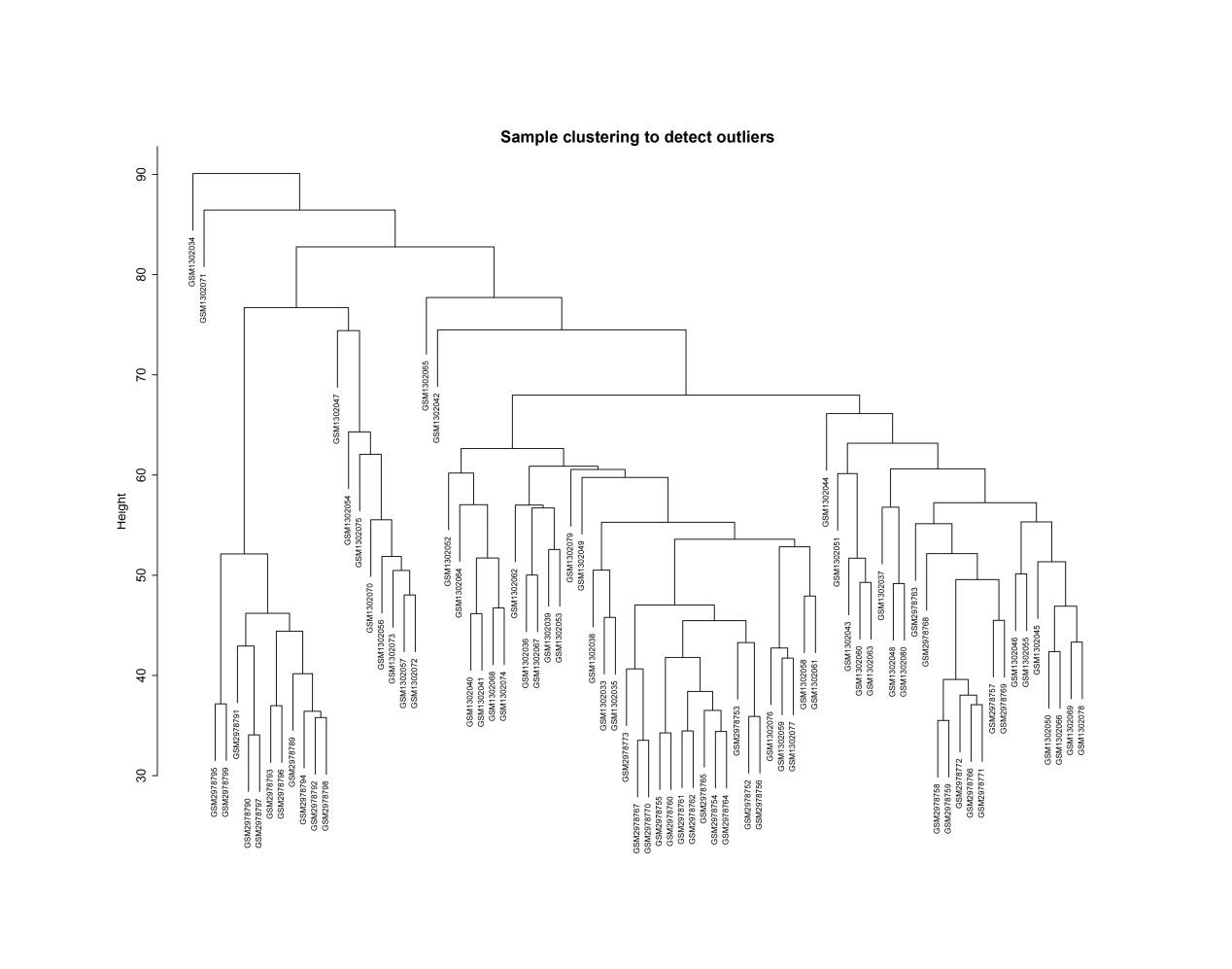


Figure Supplementary 2 The outliers GSM1302034 and GSM1302071 were excluded by cluster tree diagram
